# A qualitative process evaluation of a community conversation intervention to reduce stigma related to lower limb lymphoedema in Northern Ethiopia

**DOI:** 10.1186/s12913-022-08335-1

**Published:** 2022-08-16

**Authors:** Abebayehu Tora, Mersha Kinfe, Oumer Ali, Asrat Mengiste, Abdulkadir Ahimed, Abebaw Fekadu, Gail Davey, Maya Semrau

**Affiliations:** 1 Department of Sociology, Wolaita Sodo University, Wolaita Sodo, Wolaita Sodo, Ethiopia; 2 Center for Innovative Drug Development and Therapeutic Trials for Africa (CDT- Africa), Addis Ababa University, Addis Ababa, Ethiopia; 3 Centre for Global Health Research, Brighton and Sussex Medical School (BSMS), Brighton, UK; 4 Armauer Hansen Research Institute, Addis Ababa, Addis Ababa, Ethiopia; 5 School of Public Health, Addis Ababa University, Addis Ababa, Addis Ababa, Ethiopia

**Keywords:** Lymphoedema, Stigma, Community conversations

## Abstract

**Background:**

Lower limb lymphoedema (swelling of the lower leg) due to Neglected Tropical Diseases (NTDs) such as podoconiosis, lymphatic filariasis and leprosy is common in Ethiopia, imposing huge burdens on affected individuals and communities. Stigma significantly increases the disease burden and acts as a major barrier to accessing lymphoedema care services. A multi-component stigma reduction intervention was implemented in Northern Ethiopia. Community Conversation (CC) was one of the components implemented, and aimed to reduce stigma and enhance access to and uptake of integrated lymphoedema care services with the active engagement of community members.

**Methods:**

A cross-sectional qualitative process evaluation was conducted to document lessons focusing on CC’s relevance, outcomes and implementation challenges. Data were collected from a total of 55 purposively selected participants (26 from the CC intervention site and 29 from the control site) through key informant interviews, in-depth individual interviews and focus group discussions.

**Results:**

Community Conversations increased acceptability of health messages about lymphoedema and created peer learning opportunities for unaffected community members. Improvement in the awareness of CC participants about the causes, prevention and treatment of lymphoedema contributed significantly to the reduction of stigmatizing attitudes and discriminatory behaviors, thereby improving access to and utilization of lymphoedema care services provided through primary health care facilities. However, a range of challenges affecting implementation of CC and outcome quality were identified, including perceived complexity of the facilitation guide among facilitators, expectation of incentives among CC participants, inadequate implementation of facilitation principles and procedures, inadequacy of supportive supervision, and low engagement of untrained health workers in CC.

**Conclusions:**

With these challenges addressed, the implementation of CC integrated with other lymphoedema care services shows potential to reduce stigma and promote access to lymphoedema care services.

**Supplementary Information:**

The online version contains supplementary material available at 10.1186/s12913-022-08335-1.

## Background

Lower limb lymphoedema (swelling of the lower leg) due to Neglected Tropical Diseases (NTDs) such as podoconiosis, lymphatic filariasis (LF) and leprosy is common in tropical and subtropical areas. At the global level, the burden of lymphoedema attributable to these conditions is estimated to be 16 million [1], 4 million [2], and 200,000 [3], respectively. Of the total country burden of lymphoedema in Ethiopia, podoconiosis accounts for 64.8%, leprosy for 12.8%, and LF for 13.2% [4]. Lymphoedema imposes huge burdens on affected individuals and communities in terms of depression [5], disability [6], mental distress [7], stigma [8–11] and loss of economic productivity [12–14]. When taking these consequences into account, disease burden is even higher, at least double. Stigma is one of the key issues that significantly increases the disease burden [15], and acts as a major barrier to accessing lymphoedema care services [16, 17]. Lymphoedema-related stigma affects both health-seeking behaviour and achieving of effective treatment: in order to avoid negative reactions from others, lymphoedema patients often conceal their condition, and as a result, their symptoms may worsen [9]. The shame surrounding the disease also deters those with lymphoedema from seeking care from potentially prejudiced health workers [18]. Studies have revealed that lymphoedema patients may commonly be disqualified from full social acceptance, marginalized from participation in social affairs, discriminated against in mate selection and marriage, and have little chance of decision making and leadership roles in the community [9, 10]. Implementing and donor actors now widely recognize that joint approaches to reduce stigmatization across NTDs may be feasible given the similarities in causes, manifestations and interventions [11], but there remains a knowledge gap in regard to relevant, evidence-based stigma reduction interventions for use within integrated Morbidity Management and Disability Prevention (MMDP) programmes for lower limb lymphoedema.

Multi-component interventions have been shown to be more effective than single-component interventions for stigma reduction [19]. The World Health Organization’s (WHO) Community-Based Rehabilitation (CBR) strategy [20] can serve as a useful model, as it promotes inclusion and participation of marginalised groups through multi-sectoral interventions across five key domains (health, education, livelihood, social, and empowerment). It acknowledges that programmes need to go beyond the health domain; it empowers affected persons to take an active role in their development; it sits within a human rights framework; and it supports equity in services by building capacity amongst affected persons and their communities [21]. Due to its bottom-up approach, gender issues – important, as women are more heavily stigmatised than men [22] – and the cultural underpinnings of stigma, can be integrated within it. However, little effort has been made so far to address the stigma related to lower limb lymphoedema through multi-component interventions in the context of podoconiosis, LF and leprosy.

In response, the EnDPoINT (Excellence in Disability Prevention Integrated across NTDs) project was set up with the aim of developing, standardizing, integrating and scaling-up MMDP care services for three highly stigmatizing NTD conditions found in Ethiopia, namely podoconiosis, LF and leprosy [23]. EnDPoINT is an implementation research programme focusing on how best to integrate and scale up a holistic MMDP care package (including physical health, mental health and psychosocial care) into government-run primary health care units. It was implemented over three phases from 2018 to 2021: development of the care package in Phase 1; piloting of the care package in Phase 2; and scale up and integration of the care package into the primary health care system in Phase 3. Details about the EnDPoINT integrated holistic care package are available in previous reports [23, 24]. In essence, this care package constituted capacity building, program management, health education, MMDP, and socio-economic rehabilitation. The piloting and scale-up of the EnDPoINT programme was conducted in selected districts of Awi zone in the North West of Ethiopia [24–28]. During Phase 3 of EnDPoINT, a more detailed investigation into stigma reduction was enabled through the embedded IMPRESS (‘Improving access to integrated Morbidity management and disability Prevention Services through Stigma reduction for people with lower limb lymphoedema in Ethiopia’) project. IMPRESS adopted a multi-component interventions approach going beyond clinical management of lymphoedema. The WHO-recommended CBR model guided the identification of integrated stigma reduction components promoting the inclusion and participation of stigmatized groups across the five key domains listed above [20], which were included in the EnDPoINT holistic care package. These multi-component interventions were considered to address three major sources of stigma [9]: (i) misinformation amongst the community, patients and their families about the diseases’ causes, treatment and prevention; (ii) the common poverty and reduced quality of life due to affected individuals’ lost economic productivity; and (iii) the economic burden related to the costs of care, including transport to health facilities. Strategies to address these have been respectively: (i) educational interventions providing standardized health information, to increase disease-related health literacy; (ii) community-based socio-economic rehabilitation/strengthening of affected individuals and their families; and (iii) providing integrated services in nearby health facilities at low or no cost for patients.

The IMPRESS study applied Community Conversation (CC) to increase disease-related health literacy at the community level, with the aim of reducing stigma and improving access to MMDP services. This is a community engagement strategy most commonly applied to address health-related stigma in low literacy and resource-constrained settings [29]. A great deal of empirical evidence supports the importance of CC in reducing stigma through enabling participants to set a plan of action, develop a sense of common purpose, overcome fear, denial and passivity and move from being passive recipients of health information to active problem solvers [30]. CC is vital for enabling healthy behaviors, facilitating timely and appropriate accessing of health services and supporting optimum treatment adherence [31, 32]. CC has been widely applied in the context of HIV/AIDS, mental health and other forms of disabilities [30]. However, little is known about its role in promotion of lymphoedema care utilization and reduction of stigma. CC has been implemented within a vertical program through a community-based organization in Northern Ethiopia [33], but our understanding of its implementation through the primary health care system is limited. Through qualitative process evaluation, this study therefore aimed to document lessons learnt during the implementation of CC through the government-run primary health care system, to understand its role in promoting lymphoedema care service utilization and in reducing stigma.

## Methods

### Study setting

**Fig. 1:**
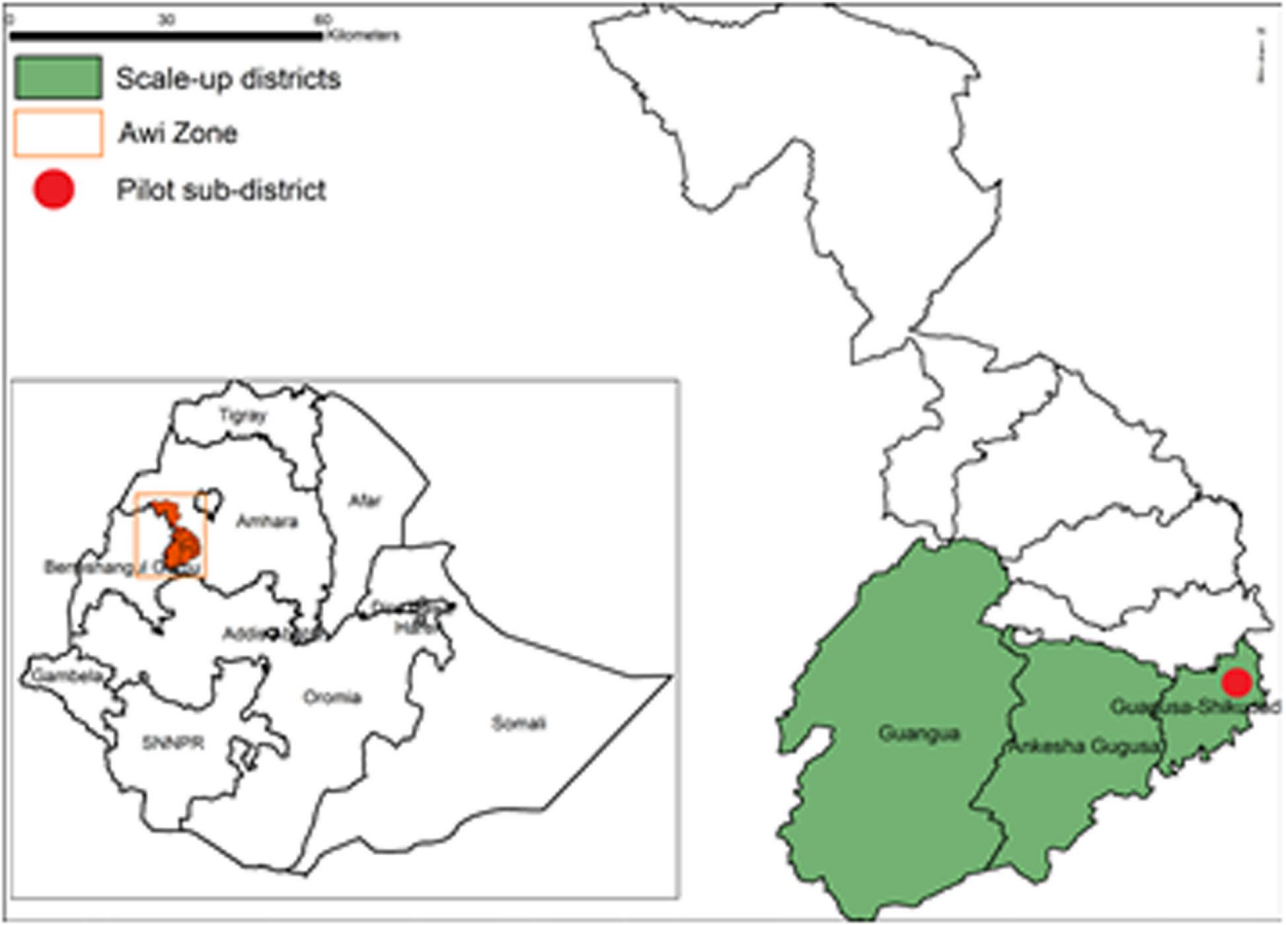
Implementation districts of the community conversation intervention

This study was conducted in Awi Zone, one of the ten zones in Amhara regional state of Ethiopia. The zone is located 469 kms north of Addis Ababa, the capital city of Ethiopia. The zone is divided into three urban and nine rural districts and covers a geographic area of 9,148 square kilometers. The elevation varies from 1,800 to 3,100 m above sea level, with an average altitude of about 2,300 m. Awi zone was selected as the study site because of the established co-endemicity of podoconiosis, LF and leprosy, and because it represented the climatic diversity found within Ethiopia. Awigna and Amharic are the main languages spoken in Awi zone. Figure 1 shows the implementation districts of the CC intervention in Awi Zone, Northern Ethiopia.

### Community conversation intervention

 A standard facilitation guide that describes the principles and procedures of health communication and that outlines health messages about the causes, treatment and prevention of lower limb lymphoedema was adapted to the study context. This was based on work by a non-governmental organization named International Orthodox Christian Charities (IOCC), which has been conducting CC as a strategy against podoconiosis-related stigma. IOCC adapted the CC facilitation guide from the HIV context and has implemented it in the West and East Gojjam zones of Ethiopia, which adjoin Awi zone. facilitation guideFacilitation guideThe CC facilitation guide consists of two major parts. The first part describes conceptual issues, objectives and expected outcomes of CC and preparatory precedures. The second part introduces three important issues: facilitation principles and procedures, CC implementation framework (that includes relationship building, problem identification, problem investigation, decision making, implementation, and reflection and review), and key competencies and tools. Basic facts on lymphoedema (with specific focus on the causes, prevention and treatment of podoconiosis) and reflective, interactive and participatory learning methods such as role play, story telling, interactive discussions and strategic questioning are outlined in the facilitation guide. Planning, performance evaluation and report formats were attached to the facilitation guide as annexes. The CC facilitation guide was originally prepared in English and was translated into Amharic, which is dominantly spoken in the study area.

Several lessons informally documented by IOCC were shared in Theory of Change workshops as part of the EnDPoINT project [23]. Based on expert suggestions in these workshops and a review of documented lessons, as part of the IMPRESS project, CC was thus added to the holistic care package of the EnDPoINT programme as one of the strategies to enhance health literacy about lower limb lymphoedema, to facilitate utilization of lymphoedema care and to reduce lymphoedema-related stigma [24].

**Fig. 2:**
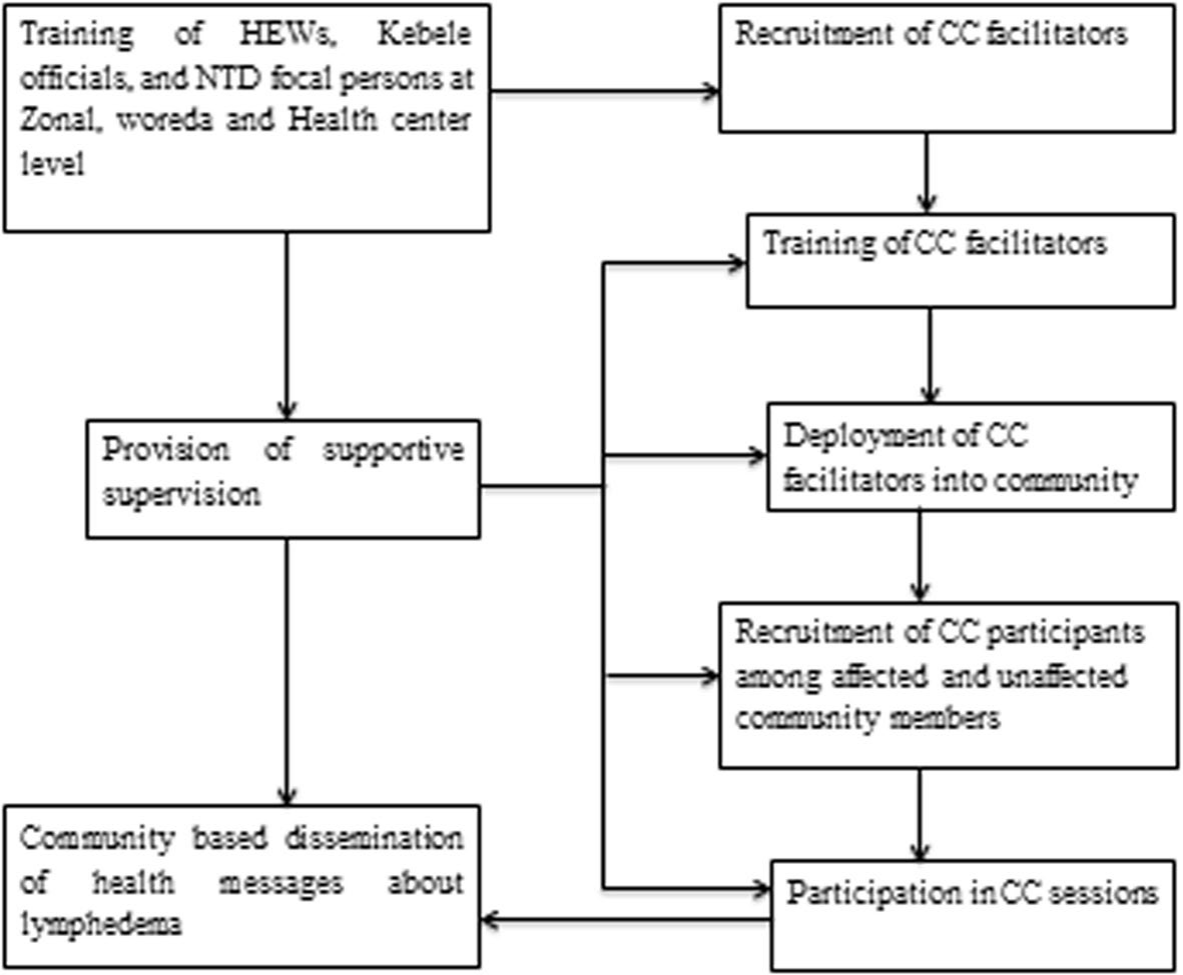
Implementation processes of community conversation intervention

The CC facilitators were recruited by Health Extension Workers (HEWs – women with one year’s training in health promotion who have responsibility for approximately 250 households in their locality) and *kebele* (lowest level administration unit) administrators using demonstrated acceptance in the community and completion of school grade 10 as criteria. HEWs and *kebele* administrators received a half-day orientation training about lymphoedema. Accordingly, they recruited religious leaders, women’s development army (WDA) leaders and lymphoedema patients whose condition had improved after treatment to serve as CC facilitators. A total of 33 CC facilitators (three from each *kebele*) were recruited from 11 *kebeles* in Guagusa Shikudad district where there are three active health centers. Using the adapted CC facilitation guide, three days’ training was provided by a CC expert to the 33 CC facilitators and NTD focal persons (three from the *woreda* – district – health office and three from health centers). Trained NTD focal persons provided support and supervision to the CC facilitators during the CC sessions. Three CC facilitators were deployed in each *kebele* to form a CC group of 30–50 participants constituting both affected and unaffected community members. A total of 400 participants engaged in the first CC session across 11 groups. Each CC group was expected to participate in two CC sessions per month. Each CC group member was expected to attend a maximum of six sessions over a three-month period. Participants were expected to disseminate health information to at least five community members per month through gatherings for various social occasions in their locality. A reporting format was attached to the CC facilitation guide, and notes of each session were reported directly to the health center NTD focal persons. The NTD focal persons provided supportive supervision to CC facilitators as they conducted CC sessions. The flow chart in Fig. 2 presents the implementation processes of the CC intervention in Awi zone, Northern Ethiopia.

The implementation of CC followed a delayed-start intervention approach to understand the added value of CC in improving access to integrated MMDP services and in reducing stigma related to lower limb lymphoedema. Of the three EnDPoINT scale up districts (Ankisha, Guangua and Guagusa Shikudad), CC was conducted in Guagusa Shikudad district for six months. Out of the three districts, Guagusa Shikudad district was selected as the CC intervention site as it served as the pilot site for the EnDPoINT project. Patients and community members might have already been exposed to general health information about lymphoedema in these districts, whereas the delayed districts were not exposed to EnDPoINT interventions at the pilot phase [24]. CC was delayed in Ankisha and Guangua districts, though other components of the EnDPoINT holistic care package continued to take place. In the delayed districts, CC was implemented after six months’ follow-up, i.e. after the study evaluation had been completed. In these districts, three days’ training was provided to CC facilitators and NTD focal persons at the *woreda* and health centers to integrate CC into routine lymphoedema care, and supportive supervision was provided for a month before the end of the project period.

### Process evaluation design

A qualitative process evaluation was conducted in Guagusa Shikudad district (where CC was implemented) and Ankisha district (one of the districts where CC was delayed). Informants from the control district were interviewed about the contributions of MMDP services in reducing stigma and the gaps that might have been addressed through CC.

### Sampling and sample size

A purposive sampling technique was employed to select study participants. Key informants for the study were *woreda* health office NTD heads, health center NTD focal persons, Health Extension Workers and the EnDPoINT field program coordinator. CC facilitators and CC participants participated in focus group discussions (FGDs) and in-depth interviews. All were purposively sampled in regard to their participation in the implementation of CC. The number of informants was determined through theoretical sampling, that is, data collection was continued until either sufficient information had been obtained or further data collection failed to generate additional themes. A total of 55 purposively selected informants (26 from the intervention site and 29 from the control site) participated in the study through key informant interviews, in-depth individual interviews and FGDs. In the intervention site, four key informant interviews, four in-depth interviews, and two FGDs (9 participants in each group) were conducted: one with CC facilitators and the other with CC groups. At the control site, there were 29 participants: two in-depth interviews, five key informants, and two FGDs (10 participants in each group) with patients and community representatives. 

### Data collection methods and tools

Qualitative data were collected over a two-week period, from May 4th -18th in 2021. Key informant interviews (KIIs), in-depth individual interviews (IDIs) and FGDs were the main methods employed. Semi-structured interview guides were developed, reviewed by all members of the research team and used during interviews. We used an iterative approach to consistently update the interview guides and further explore issues that emerged during interviews. A list of open-ended questions based on predefined themes prompted reflection about the quality of implementation (relevance, challenges), impact and outcome quality of the intervention. AT (a sociologist with over 10 years experience in qualitative research) led the interviews. MK with the assistance of local guides from the *Woreda* health office assisted recruitment of interviewees. Key informants were approached in their offices, and in-depth interview informants were approached in their homes. FGD participants were called to the nearest health facility. The transportation expenses of FGD participants were covered. The individual interviews lasted for a maximum of one hour, while FGDs lasted one-and-a-half hours. All participants could speak Amharic, so interviews were conducted in this language. All interviews were audio-recorded with permission from participants.

### Research team

The research team comprised five researchers (GD, AF, MS, OA and AT) whose extensive experience in implementation research in general and qualitative research in particular shaped the entire process of the study. The assistance of A lymphoedema expert (AM), a project coordinator (MK) and a field project coordinator (AA) contributed for successful completion of fieldwork. All interviews were led by AT with the facilitation of MK. The backgrounds of the researchers were described to all informants of the study. AT led the interviews in a private venue. AA provided supportive supervision to health extension workers and NTD focals during implementation of the CC intervention. AT and MK had no established relationship with the informants before the interviews commenced.

### Data analysis

The audio recordings were transcribed verbatim, translated into English, and imported into NVivo-11 software (QSR NVivo, QSR International, Burlington, MA, USA). A thematic qualitative data analysis method was employed to analyze the data. AT and MK led the coding while all other authors participated in reviewing the codes. Coding of data was based on the predefined themes included in the interview guides and on a grounded theory approach in which themes are inductively identified during the coding process.

## Results

Of the 26 informants who participated in the study from the intervention site, 12 were female and 14 were male At the control site, there were 29 participants, of whom 14 were female and 15 were male. The age range of informants in the intervention and control sites was 32–66 years. The majority of in-depth individual interviews and FGD informants (patients and community members) had no formal education. The educational attainment of CC facilitators participating in the focus groups ranged from basic education to grade 10.

Our findings are presented below under four predefined themes derived from the empirical literature related to relevance, implementation mechanisms and outcomes of CC in other contexts: perceived relevance of CC as health communication; outcomes of CC; added value of CC; and implementation challenges related to CC. Under each theme, several sub-themes are identified and discussed.

### Perceived relevance of CC as health communication strategy

#### Facilitating acceptability of health messages

As opposed to vertical communication of health messages through health professionals, CC was perceived to encourage peer learning and to promote acceptability of health messages. As peers can freely interact with their fellows in a language they understand, they are less likely to feel coerced to participate or be shy to ask questions.

“*Basically, CC differs from the conventional health education methods in that lay people are trained to communicate health messages. As the participants are of similar status with the facilitators, CC promotes the acceptability of the health message. In the conventional approach, it is not easy to bring the needed change as health professionals use technical terms during communication. The engagement of both patients and non-patients in the same group facilitates speedy learning behavioral change” (Key informant, lymphoedema program field coordinator).*

A CC participant also echoed the views reflected by the lymphoedema program coordinator. The participant indicated that CC plays an important role in enhancing awareness about the cause of lymphoedema and the consequent avoidance of risky behaviors. According to the IDI informant, CC has shaped the understandings and behaviors of both affected and unaffected participants in a positive way. Many were reported to use footwear and continue foot hygiene after their participation in CC. The CC participants also gained confidence to communicate health messages about lymphoedema to their neighbours in the community.

*“We had little knowledge about the cause of lymphoedema. After our participation in CC, we learned that many of our people suffered from the disease due to lack of awareness. We got a big relief since we learned barefoot exposure as a cause of many diseases. After participation in this education, we are observing changes in the behavior of many people in our community. The lymphoedema condition of patients has also improved. Many of them started to use footwear and actively participate in social affairs. We are lucky to take part in this education. We are thankful to those who brought this education to us. We are sharing this information to our fellows in social gatherings like ‘equb’ [a traditional way of saving money]” (IDI with unaffected CC participant, intervention site).*

#### Creating opportunities for learning for unaffected community members

Informants also appreciated CC for the opportunity it created for affected and unaffected community members to come together to learn about lymphoedema. Unaffected community members holding various forms of stigmatizing attitudes towards lymphoedema patients and who initially resisted being in the same session were reported to become comfortable interacting in CC sessions.

*“Both unaffected and affected persons participated in Community Conversation sessions. Initially, unaffected persons were resistant to participate in CC sessions with affected ones. But, as they got correct information about the causes of lymphoedema and saw the demonstrations and witnesses of lymphoedema patients about curability they felt happy about CC. Most of the participants who had misconceptions were later observed to be happy as we informed them that lack of hygiene and barefoot walking are major causes of lymphoedema related to podoconiosis.” (FGD participant, CC facilitator, intervention site).*

*“What is unique about CC is that it engages many stakeholders at a time. Government officials, community leaders, religious leaders and health workers are brought together in the process of disseminating health information.” (Key informant, HC focal person, intervention site).*

#### Outcomes of community conversations

The primary outcomes expected from CC were reduced misconceptions about lymphoedema, enhanced uptake and adherence with clinical and self-care, and reduced stigma. Interviews with informants provided evidence that CC plays an important role in achieving the expected outcomes.

#### Reducing misconceptions about lymphoedema

Informants indicated that misconceptions about lymphoedema were rampant at the beginning of the intervention. Through CC, participants corrected their misconceptions about the causes, prevention and treatment of lymphoedema particularly related to podoconiosis (one of the three conditions targeted in the holistic care package).

*“Community Conversation played an important role in improving the awareness of the community. Most CC groups have conducted five sessions. They had misconceptions about lymphoedema when they came to CC. There were changes after they participated in the CC sessions. When you ask those who used to say podoconiosis is caused by snake bite, they correctly mention walking barefoot as a cause.” (Key informant, HC NTD focal person, intervention site).*

Similarly, their conceptions about prevention mechanisms of lymphoedema also improved. Participants in CC sessions recalled what the facilitators taught them about prevention of podoconiosis.

*“I recall that they advised us to keep the hygiene of our children. They said the disease is not hereditary and can be prevented through consistent use of footwear. Even though we cannot avoid contact with soil as farmers, we started to wash our feet immediately.” (FGD with CC participant, intervention site).*

*“I used to walk barefoot and never used shoes. After they [CC facilitators] informed us about the cause of the disease, I started to use footwear.” (FGD with CC participant, intervention site)*.

#### Enhancing adherence with clinical lymphoedema care

Health center focal persons indicated an improvement in the number of lymphoedema patients seeking care after the implementation of CC. The improvement in awareness of patients about the availability of treatment seemed to boost the confidence of patients about lymphoedema care services provided through health centers.

*“The correction of misconceptions about treatment of lymphoedema through Community Conversations increased the number of patients seeking care in our health center. In the first session, most of the participants were not aware of the availability of treatment. The follow up of patients has increased after their participation in Community Conversation sessions. I strongly believe that Community Conversation has broken the rampant misconceptions.” (Key informant, HC NTD focal person, intervention site).*

#### Promoting self-care practice

One of the reported outcomes of CC was its capacity to promote self-care among lymphoedema patients.

*“Patients get the chance to learn from other participants of CC who have similar conditions. This helps them learn how to practice self-care at their homes.” (Key informant, HC NTD focal person, intervention site).*

#### Reducing stigma and discrimination

Most informants emphasised the severity of stigma and discrimination in the community, attributing it to widely held misconceptions about the cause, prevention and treatment of lymphoedema. Self-stigma among patients was reported to reduce after participation in CC sessions.

*“Since learning about the importance of hygiene during Community Conversation sessions, patients’ practice of hygiene has improved and their confidence in social interaction has increased. Before this, bad smells related to their condition repelled people from interaction with patients as they did not keep their foot hygiene adequately.” (Key informant, HC NTD focal person, intervention site).*

Informants also suggested that improved understandings about the cause of lymphoedema helped community members to avoid prejudice and discrimination against patients.

*“They [CC facilitators] convened us at the health post every Sunday once in a month. They advised us not to discriminate against lymphoedema patients. We also refrained from doing so.” (FGD with unaffected CC participant, intervention site).*

*“We used to see lymphoedema patients as full of wounds and belittle them. But, when we witnessed improvement after they received treatment and after we heard that the disease is not contagious, we don’t feel discomfort to interact with them anymore.” (FGD with CC participant, intervention site).*

*“After the implementation of Community Conversations, the social acceptance of lymphoedema patients has improved. After unaffected community members learned that the disease is not contagious, they started to feel free to participate in social affairs with affected persons.” (FGD participant, CC facilitator, intervention site).*

#### Added value of community conversations

To determine the added value of Community Conversations, the manifestations of misconceptions and stigmatizing attitudes among informants from control sites were compared to the intervention site. At control sites, both patients and community members were expected to hold higher levels of misconceptions and stigmatizing attitudes, as more attention was given to physical and mental health care through MMDP services in the control sites. Only general health information was provided to patients and community represenatives about lymphoedema compared to the CC intervention site where a structured health education campaign was conducted. Interviews with informants from control sites suggested that general dissemination of messages about lymphoedema to community representatives and patients was not adequate in addressing misconceptions and stigmatizing attitudes. This was evident in group interviews held with the community. In regard to the cause of lymphoedema related to podoconiosis, informants mentioned heredity and contagion.

*“Podoconiosis is common in our area. It is hereditary. I know siblings who were affected by the disease.” (FGD participant, community representative, control site).*

*“Podoconiosis is caused by stepping into the water that an affected person used to wash his/her feet. It can also be caused by sharing of shoes used by an affected person. So, those in the same family should use things separately.” (FGD participant, community representative, control site).*

*“When people walk barefoot, they step on residues which cause swelling. Flies also transmit the disease from a patient with wounds on his feet.” (FGD participant, community representative, control site).*

Some participants did correctly link barefoot walking in soil as a cause of podoconiosis. However, they did not properly recognize how barefoot exposure causes the disease.

*“Since our people walk barefoot, they are exposed to many toxic things in the soil. These things enter into the foot as we walk barefoot and cause lymphoedema.” (FGD participant, community representative, control site).*

The aforementioned misconceptions were also apparent in the stigmatizing attitudes of non-affected community members in the control site. Some informants indicated that they feared interacting with patients, perceiving the disease to be contagious.

*“Yes, there is fear and exclusion in the community. People think that the disease is contagious.” (FGD participant, community representative, control site).*

*“As we think the disease is hereditary, we are not happy to allow inter-marriage with an affected family. They also don’t feel confident to get into marriage with an unaffected family.” (FGD participant, community representative, control site).*

*“There are times we observe that unaffected people fear to sit beside us. I think it is because of lack of information. But, I don’t give due attention in such circumstances. I just ignore it.” (IDI informant, lymphoedema patient, control site).*

However, some informants perceived a reduction in lymphoedema-related stigma in the control site, associating it with utilization of clinical care. Observing an improvement in the physical condition of lymphoedema patients, their confidence to interact with community members increased.

*“Earlier, the patients felt ashamed to sit besides non-affected people. Unaffected people also felt discomfort to sit besides them thinking that flies sitting on them can transmit the disease. However, after they started getting treatment, such feelings reduced because of improvement in their physical condition.” (FGD participant, community representative, control site).*

### Implementation challenges related to community conversations

#### Perceived complexity of the facilitation guide

CC facilitators and Health Center focal persons were asked about the user-friendliness of the CC facilitation guide. There were mixed perceptions, some informants a findingthe content of the facilitation guide difficult, others associating any difficulties with their own low literacy, and yet others blaming the lack of preparedness of facilitators. According to a Health Center focal person, most sections in the facilitation guide focussed on principles of CC implementation, and failed to address lymphoedema conditions in sufficient depth.

*“The CC facilitation guide focuses mainly on CC principles rather than messages. It does not go into details of lymphoedema conditions. That is why I think CC facilitators faced difficulty communicating health messages as expected.” (Key informant, HC focal person at intervention site).*

The presence of technical terms in English was thought to make the facilitation guide difficult to understand.

*“The English words present here and there in the facilitation guide are difficult to understand. Though I can read them, it is challenging for me to translate technical terms on podoconiosis.” (FGD participant, CC facilitator, intervention site).*

CC facilitators in the FGDs thought that their own low levels of education were the main problem.

*“The facilitation guide given to us is very difficult to understand. I cannot say we have applied CC sessions as expected in the facilitation guide. I think our level of literacy limited our understanding of the facilitation guide. The reporting formats were also confusing.” (FGD participant, CC facilitator, intervention site).*

According to another informant, the difficulty of the CC facilitation guide was not related to its content. The low motivation of some CC facilitators to read the facilitation guide in depth before each CC session was raised as a challenge.

“Some CC facilitators did not make enough effort to read the facilitation guide. They conducted sessions without properly reading the facilitation guide. (FGD participant, CC facilitator, intervention site)

#### The expectation of incentives among CC participants

The expectation of incentives among CC participants was perceived to be a challenge for the implementation of CC. Most of the CC facilitators indicated that the number of CC participants decreased as CC sessions continued, due to a lack of incentives.

*“During the first day of CC sessions, all invited participants appeared. But, at the next sessions, their number decreased. When we asked them why some participants are missing, they complained about a lack of per diem. They were not interested in participating. We managed to conduct the expected number of sessions with the support of kebele officials and Health Extension Workers who continued to insist community members to participate.” (FGD participant, CC facilitator, intervention site).*

*“The facilitators strive as much as they can. But, there is dropout among participants. Patients particularly ask for shoes promised to be provided to them as they continue receiving treatment. They expect us to provide them with shoes.” (Key informant, HC focal person, intervention site).*

The decrease in the number of participants was reported to be high for non-affected community members who showed low interest due to a lack of incentives.

*“The unaffected community members were less interested. They require some amount of money for refreshments. It is with repeated insistence that they come to the CC venue.” (FGD participant, CC facilitator, intervention site).*

#### Low commitment of CC facilitators to consistently run CC sessions

Once deployed into their communities, all the facilitators were expected to facilitate CC sessions. Some facilitators were unwilling and others effectively disappeared. Some facilitated the CC sessions out of respect or fear of the Health Center focal persons or *kebele* officials.

*“One of the challenges for the implementation of CC is that some of the CC facilitators were unwilling to facilitate the CC sessions. It was we who insisted that they do so. One of the CC facilitators disappeared.” (Key informant, HC focal person, intervention site).*

#### Inadequate implementation of CC principles and procedures

Some of the CC facilitators were reported to face difficulties in following the principles and procedures in the facilitation guide while running sessions. They were found to simply deliver health messages without properly ensuring whether these messages were accepted and understood among participants.

*“It is very difficult for them [CC facilitators] to follow the principles and procedures of CC. They head directly to delivering messages. They needed our assistance in most cases. They cover all messages to be delivered in different sessions at once, sketchily. They don’t go into details. If we leave them alone to conduct the CC, the CC they conduct is incomplete.” (Key informant, HC focal person, intervention site).*

Some of the facilitators suggested the need for additional training on the implementation of CC to enhance their competency on the proper use of the facilitation guide. The importance of experience-sharing opportunities was also stressed by CC facilitators so that they could learn from other CC facilitators.

*“We would like to have more training on the CC facilitation guide and its implementation. We want to be role models to other woredas.” (FGD participant, CC facilitator, intervention site).*

*“We would appreciate if experience-sharing opportunities were created. We could review the strengths and weaknesses of our performance and learn from others.” (FGD participant, CC facilitator, intervention site).*

#### Inadequacy of supportive supervision

*Woreda* and Health Center NTD focal persons, Health Extension Workers and *kebele* administrative officials were expected to engage in supportive supervision. However, when asked about the adequacy of supportive supervision, informants indicated gaps. The engagement of the stated actors was thought to be inadequate. Some Health Center NTD focal persons complained that the CEOs of their health centers did not cooperate with either sparing them other clinical duties or allowing them to use vehicles to get to the health posts.

*“When we wanted to visit health posts for supportive supervision, the CEO of this health center refused to allow us to use the motorbike. We were told to go there on foot. Only after tough discussion could we even transport lymphoedema care kits. There are patients with severe stage disease who need house visits as it is difficult for them to walk to the health centers.” (Key informant, HC focal person, intervention site).*

The involvement of Health Extension Workers was found to be limited in supporting CC facilitators with the recruitment of participants. Their assistance with the facilitation of CC sessions was suggested.

*“Apart from connecting CC facilitators with participants, they are not actively engaged in the implementation of CC. The CC facilitators prefer to contact us for support, though Health Extension Workers could provide the needed support. Health Extension Workers have not yet fully owned CC as part of their duty. They did not adequately supervise the facilitators. They didn’t participate in the three days’ training. This may be the reason.” (Key informant, HC focal person, intervention site).*

#### Low engagement of health workers other than trained focals

Health center NTD focal persons indicated that untrained health workers in the health facility consider the management and supervision of lymphoedema care services and CC only to be the duty of health workers who participated in lymphoedema and CC training workshops. Though the trained health workers indicated that they had provided a one-day orientation on lymphoedema care and CC to all health workers, that was not adequate enough to make them feel equally accountable for the care of lymphoedema patients and supervising CC groups.

*“Only trained health center focals feel responsibility to provide the services. Other health workers don’t feel equal responsibility. When we leave the health center for outreach visits, other health workers don’t feel accountable to take care of lymphoedema patients.” (Key informant, HC NTD focal person, intervention site).*

*“To tell you frankly, other health workers are not actively engaged in providing lymphoedema care apart from the trained ones like us. Though they received orientation, they don’t feel confident. They call us when lymphoedema patients visit the health center. Community Conversations will also face the same challenge. Other health workers should also participate in the CC training for it to be implemented properly.” (Key informant, HC NTD focal person, intervention site).*

## Discussion

The role of CC in health education and promotion has been widely recognized. However, existing evidence supports the implementation of CC as standalone program by development partners, mainly in the context of HIV/AIDs [29–32]. Little empirical evidence is available on the role of CC integrated into the primary health care system as stigma reduction strategy in the context of lymphoedema care. Through the EnDPoINT/IMPRESS program, CC were conducted with the aim of promoting optimum utilization of integrated holistic lymphoedema care services through reducing stigma. This qualitative process evaluation explored the added value of CC in reducing stigma and promoting access to lymphoedema care services provided through the primary health care system focusing on its relevance, outcomes and implementation challenges.

The relevance of CC as lymphoedema-related stigma reduction strategy was recognized by all of the informants including *Woreda* and health center NTD focals, patients, community members, and health extension workers. Through empowering low-literacy rural people to be active agents of health communication, CC was perceived to be a good alternative to reach rural people whose access to health information about lymphoedema is compromised by stigmatizing views among both health workers and community members at large [18]. In the present study, CC was also perceived to help overcome the logistic and infrastructural challenges faced by health facilities expressed in limiting the active engagement of health workers in community-based health education. What is more, the participation of both affected and unaffected community members in CC sessions created opportunities for peer learning about the lived experience of lymphoedema patients and care outcomes. Congruent with a previous report [30], health workers and facilitators considered CC to increase the acceptability of health messages among community members who had high levels of misconceptions and stigmatizing attitudes.

More importantly, this qualitative process evaluation demonstrated the potential of CC, integrated into the primary health care system, to improve access to lymphoedema care through addressing misconceptions and reducing stigma. With the participation of more lymphoedema patients, clinical follow up and self-care practices have reportedly been improved since the commencement of CC in the study area. The high levels of misconceptions that patients and community members held about the causes, prevention and treatment of lymphoedema were addressed. These stigmatizing views and discriminatory behaviors against lymphoedema patients were reduced. These outcomes demonstrate the acceptability of implementing CC using existing infrastructure and human resources within the government-run primary health care system. These positive outcomes are in line with the reports of other studies that have documented a number of success stories of CC in other disease contexts such as HIV/AIDS [30, 31], mental health [34], and child health service utilization [35]. A recent study also suggested the need to overcome lymphoedema-related stigma for improving care for cancer-related and other forms of lymphoedema in low and middle income countries [36].

However, a range of challenges that affect the implementation of CC through the primary health care system were identified in this study. Some of the facilitators of CC were challenged to properly use the facilitation guide and follow communication principles and procedures. This was attributed partly to low literacy expressed as limited analytical and conceptualization skills. Facilitators suggested that a three-day training workshop was inadequate and recommended review meetings to provide opportunities for learning from strengths and weaknesses encountered. The literature provides mixed evidence regarding the optimum number of days for CC training workshops. According to Lemma and colleagues, a complete grasp of the training content will take three days of training time [37]. In another study, a CC training workshop was extended to 10 days [38]. A gap in our study could be the direct adoption of a CC facilitation guide developed and implemented by a development partner. As a vertical program, development partners allocate resources and time [38], which government-run health facilities may not be able to afford. The CC facilitation guide adopted from development partners may therefore need to be tailored to the context of CC facilitators recruited and monitored by health workers in the primary health care facilities. Morever, the lack of objective indicators in the facilitation guide limited our understanding of signs of change due to the intervention. In addition to the facilitation guide, the use of tools like posters, pictures, and a pre-post test question template with objective indicators of change could also have assisted the CC facilitators to properly conduct the CC sessions, as suggested by other studies [37].

The CC facilitators reported supportive supervision by health workers to be inadequate. NTD focal persons at the health center also raised bureaucratic and logistic challenges related to supportive supervision of CC sessions. One of the bureaucratic challenges was low interest of some CEOs of health facilities to prioritize lymphoedema services, meaning limited access to vehicles or assignment of other duties to NTD focals on the days scheduled for community outreach visits. This perhaps can be attributed to logistical challenges at health facilities and the small number of health workers trained on lymphoedema. Health extension workers could have played a significant role in filling the supervision gap. However, the engagement of health extension workers was limited to identification and recruitment of CC facilitators. As health extension workers did not participate in the three-day CC training, they considered assisting the CC facilitators to be the duty of those who had taken part. Though other health workers received a general orientation about lymphoedema at the facility level, they were also reported to perceive lymphoedema care services to be the duty of those health workers who had received the training. The inclusion of more health workers in CC training workshops would provide more opportunities for closer supportive supervision. A previous study also suggested the closer follow-up of facilitators through supervision or review meetings to be important for the successful implementation of CC [38]. The CC facilitators needed technical support on the use of the facilitation guide, communication skills and handling of queries from participants. They faced difficulties following the procedures and principles in the facilitation guide, and promoting the acceptability and practice of recommended preventive behaviors. As the monitoring and performance evaluation tools were not adequately utilized in each session, it was difficult to objectively assess the early signs of change observed among participants, which would have enabled more objective judgement of the outcomes of CC [37].

Another challenge was the lack of incentives for CC facilitators and participants. Unfulfilled expectations around incentives and refreshments, particularly from participants, were reflected in inconsistent attendance at CC sessions, dropouts being common in subsequent sessions. The commitment of facilitators to strictly follow and implement CC principles and procedures and to conduct house visits to ensure acceptance and practice of healthy behaviors was limited. As an integrated program, logistical issues were expected to be addressed through the health system and community participation. However, the allocation of financial and material resources for the implementation of CC through the health system was not prioritized. Coupled with lack of financial incentives, expectation of benefits has been reported by others to be a challenge when implementing community-based interventions through the health system [39]. Government bodies and development partners must address the logistic and financial challenges experienced by primary health care facilities when implementing interventions like this. Arranging motivation mechanisms including review meetings, material and financial incentives for CC facilitators might strengthen their sense of ownership and commitment. In this regard, development partners can play a significant role until CC is fully integrated into the health system as lymphoedema-related stigma reduction strategy.

### Limitations and future research directions

This qualitative process evaluation is not without limitations. As data reported in this study are qualitative, the reliability of differences observed in the intervention and control sites in terms of outcomes may be questionable. a. To address this issue, we conducted a quantitative pre-post evaluation of the impacts of the CC in both control and intervention arms, which will be reported subsequently. The use of mixed methods for process evaluation of community-based interventions may enhance the validity and reliability of outcomes. For instance, this report was based only on the data obtained through interviews held with participants, facilitators and health workers. The qualitatative process evaluation could be substantiated by the use of structured monitoring tools that would help documentation of observed changes among participants’ attitudes and behavior in each CC session. Gender differences in the composition of the key informant and end user groups reflect wider societal gender biases, and are difficult to correct when very few heads of health centres or NTD leads are female.

## Conclusions

In conclusion, even though there was a range of observed challenges, the CC intervention was found to play an important role in promoting access to integrated holistic lymphoedema care services through addressing misconceptions and reducing stigma. Apart from serving as inputs for further improving the quality of the CC implementation process, the lessons documented through this qualitative process evaluation could help researchers, development partners and policy makers to optimize the benefits of integrating CC into the primary health care system as a feasible health communication strategy in the context of stigmatizing disease conditions like lymphoedema [5, 14, 25, 26].

## Supplementary Information

**Additional file1. **Consolidated criteria for reporting qualitative studies (COREQ): 32-item checklist

## Data Availability

All data generated or analysed during this study are included in this published article.
