# Supplementary material for: A qualitative process evaluation of a community conversation intervention to reduce stigma related to lower limb lymphoedema in Northern Ethiopia
Source: BMC Health Serv Res. 2022 Aug 16;22:1043. doi: 10.1186/s12913-022-08335-1 (PMC9380383; doi:10.1186/s12913-022-08335-1)
Supplement: Supplementary file 1 — Additional file1. Consolidated criteria for reporting qualitative studies (COREQ): 32-item checklist [file 12913_2022_8335_MOESM1_ESM.docx]

**Consolidated criteria for reporting qualitative studies (COREQ): 32-item checklist**

Domain 1: Research team and reﬂexivity

.............................................................................................................................................................................

Personal Characteristics

1. Interviewer/facilitator: Which author/s conducted the interview or focus group?

AT conducted the interview with the assistance of MK (see lines 247 and 248).

1. Credentials: What were the researcher’s credentials? E.g. PhD, MD

AT (PhD)

1. Occupation: What was their occupation at the time of the study?

AT (Academician and researcher at a public university in Ethiopia)

1. Gender: Was the researcher male or female?

AT (Male)

1. Experience and training: What experience or training did the researcher have?

AT has over 10 years of experience in qualitative research.

Relationship with participants:

1. Relationship established: Was a relationship established prior to study commencement?

AT had no established relationship with the informants prior to study commencement.

1. Participant knowledge of the interviewer: What did the participants know about the researcher? e.g. personal goals, reasons for doing the research

Participants were fully informed about the purpose of the study during reading of the consent information that includes information about the researcher’s institution and that of the collaborating institution.

1. Interviewer characteristics: What characteristics were reported about the interviewer/facilitator? e.g. Bias, assumptions, reasons and interests in the research topic

AT is a sociologist with over 10 years of experience in qualitative research (see lines 247 and 248).

Domain 2: study design

………………………………………………………………………………………………………….

Theoretical framework

9. Methodological orientation and Theory: What methodological orientation was stated to underpin the study? e.g. grounded theory, discourse analysis, ethnography, phenomenology, content analysis

Both thematic (themes derived from empirical literature) and grounded theory approaches were used to analyze the data (see lines 264-270).

Participant selection

1. Sampling: How were participants selected? e.g. purposive, convenience, consecutive, snowball

Purposive sampling method was employed to select informants.

1. Method of approach How were participants approached? e.g. face-to-face, telephone, mail, email

The participants were approached face-to-face.

1. Sample size: How many participants were in the study?

A total of 55 informants participated in the study (see lines 232-240)

1. Non-participation: How many people refused to participate or dropped out? Reasons?

All of the participants were willing to participate.

Setting

1. Setting of data collection: Where was the data collected? e.g. home, clinic, workplace

Both at workplace and at home (see lines 255-259).

1. Presence of non-participants: Was anyone else present besides the participants and researchers?

Not at all.

16. Description of sample:What are the important characteristics of the sample? e.g. demographic data, date

Gender and educational attainment informants described in lines 280-291.

Data collection

17. Interview guide: Were questions, prompts, guides provided by the authors? Was it pilot tested?

Yes, we have provided interview guides as supplementary file during submission of revision.

18. Repeat interviews: Were repeat interviews carried out? If yes, how many?

No, we haven’t conducted repeated interviews.

19. Audio/visual recording: Did the research use audio or visual recording to collect the data?

Yes, we used audio recorder during interviews.

20. Field notes: Were ﬁeld notes made during and/or after the interview or focus group?

Yes, field notes were made during interviews.

21. Duration: What was the duration of the interviews or focus group?

The interviews lasted a maximum of one and half hours.

22. Data saturation: Was data saturation discussed?

Yes data saturation was discussed.

23. Transcripts returned: Were transcripts returned to participants for comment and/or correction?

We haven’t returned the transcripts to informants as the interviews were conducted face to face in which notes were verified on the setting.

Domain 3: analysis and ﬁndings

……………………………………………………………………………………………………………………

Data analysis

24. Number of data coders: How many data coders coded the data?

AT, MK, and MS (three of the authors) coded the data.

25. Description of the coding tree: Did authors provide a description of the coding tree?

Yes, the description of the coding tree included in analytical memo.

26. Derivation of themes: Were themes identiﬁed in advance or derived from the data?

Themes were derived both in advance and from the data.

27. Software: What software, if applicable, was used to manage the data?

NVIVO version 11 software was used to manage and analyze the data.

28. Participant checking: Did participants provide feedback on the ﬁndings?

We checked with participants our understanding of the data during interviews through confirmatory questions.

Reporting

29. Quotations presented: Were participant quotations presented to illustrate the themes /ﬁndings? Was each

quotation identiﬁed? e.g. participant number

Yes we provided quotations with identifiers.

30. Data and ﬁndings consistent: Was there consistency between the data presented and the ﬁndings?

Yes.

31. Clarity of major themes: Were major themes clearly presented in the ﬁndings?

Yes.

32. Clarity of minor themes: Is there a description of diverse cases or discussion of minor themes?

Yes.
